# Maximizing Nanosatellite Throughput via Dynamic Scheduling and Distributed Ground Stations

**DOI:** 10.3390/s25247538

**Published:** 2025-12-11

**Authors:** Rony Ronen, Boaz Ben-Moshe

**Affiliations:** School of Computer Science, Ariel University, Ariel 4070000, Israel; benmo@g.ariel.ac.il

**Keywords:** nanosatellites, new space, wireless communication systems, satellite Internet of Things, resource allocation, optimization, LoRa

## Abstract

Nanosatellites in Low Earth Orbit (LEO) are an attractive platform for commercial and scientific missions, but their downlink capacity is constrained by bandwidth and by low ground station duty cycles (often under 5%). These limitations are particularly acute in heterogeneous cooperative networks, where operators seek to maximize “good-put”: the number of unique messages successfully delivered to the ground. In this paper, we present and evaluate three complementary algorithms for scheduling nanosatellite passes to maximize good-put under realistic traffic and link variability. First, a Cooperative Reception Algorithm uses Shapley value analysis from cooperative game theory to estimate each station’s marginal contribution (considering signal quality, geography, and historical transmission patterns) and prioritize the most valuable upcoming satellite passes. Second, a pair-utility optimization algorithm refines these assignments through local, pairwise comparisons of reception probabilities between neighboring stations, correcting selection biases and adapting to changing link conditions. Third, a weighted bidding algorithm, inspired by the Helium reward model, assigns a price per message and allocates passes to maximize expected rewards in non-commercial networks such as SatNOGS and TinyGS. Simulation results show that all three approaches significantly outperform conventional scheduling strategies, with the Shapley-based method providing the largest gains in good-put. Collectively, these algorithms offer a practical toolkit to improve throughput, fairness, and resilience in next-generation nanosatellite communication systems.

## 1. Introduction

Satellite communication constitutes a cornerstone of the global communications infrastructure, offering vital support to an array of rapidly advancing terrestrial technologies, including the Internet of Things (IoT), smart cities, and electric vehicles. Within this dynamic environment, Low Earth Orbit (LEO) nanosatellites have risen to prominence. Their appeal is driven by substantial reductions in development cost, miniaturization of components, and advances in on-board computing power. Compared to their geosynchronous counterparts, nanosatellites offer advantages in deployment flexibility and low communication latency, making them highly suitable for emerging applications such as remote sensing, Earth observation, and commercial telecommunications.

Despite their inherent advantages, LEO nanosatellites, which travel at speeds exceeding 27,000 km/h, face a critical operational limitation: severely constrained downlink opportunities. A reliance on a single, dedicated ground station (GS) typically permits contact only 1–4 times daily, with each pass lasting mere minutes. This results in an exceptionally low duty cycle, often less than 1% of a 24-h period, which significantly restricts the volume of data that can be collected and transmitted. Compounding this challenge, the establishment and maintenance of traditional dedicated GS facilities involve substantial financial and regulatory hurdles. To mitigate these restrictions and accommodate the burgeoning demand for nanosatellite data, distributed, open-source ground station networks have emerged as essential, cost-effective solutions for tracking and communicating with these platforms. Initiatives such as SatNOGS [1] and TinyGS [2] leverage the global community to deploy widespread, low-cost receivers. These collective networks effectively transform the operational model, providing greater coverage and increased data throughput potential [3].

While distributed networks successfully increase the number of potential downlink contacts, a significant technical inefficiency persists in the scheduling and resource allocation protocols. The current operational paradigm often employs a greedy allocation strategy, where each ground station independently prioritizes the nanosatellite pass with the earliest Acquisition of Signal (AOS). This locally optimal approach inevitably leads to a crucial network-level failure: message redundancy. High-power or frequently visible nanosatellites are often received simultaneously by numerous stations, wasting collective bandwidth. Conversely, lower-power or less-visible nanosatellites are frequently ignored. This diminishes the network’s overall good-put, defined as the total number of unique messages successfully transmitted and received. The fundamental problem addressed by this study is the development of a scalable, distributed resource allocation strategy that shifts the objective from maximizing local contact time to maximizing network-wide unique message reception, thereby ensuring efficient utilization of the cooperative ground station infrastructure.

In this paper, we present a novel resource allocation optimization framework explicitly designed for distributed, ad hoc, and heterogeneous LEO nanosatellite networks. Our methodology diverges from traditional centralized scheduling by prioritizing the global good-put, the expected number of unique messages received across the entire network, by dynamically managing assignment conflicts and redundancy. The primary contributions are the formulation of the unique message maximization problem and the proposal of a set of complementary, efficient scheduling algorithms:

- **Cooperative Reception Algorithm:** An algorithm designed to quantify the marginal contribution of each ground station to the network’s unique message reception using principles derived from cooperative game theory, ensuring fair and efficient resource assignment.
- **Pair Utility Optimization:** An approach focusing on dynamically refining assignments through localized, pairwise utility comparisons between competing ground stations to mitigate biases and adapt swiftly to changing orbital conditions and network load.
- **Weighted Algorithm:** A method utilizing a message pricing and weighted bidding mechanism to allocate resources optimally by maximizing the expected reward (unique message capture) across the entire cooperative network.

**Figure 1:**
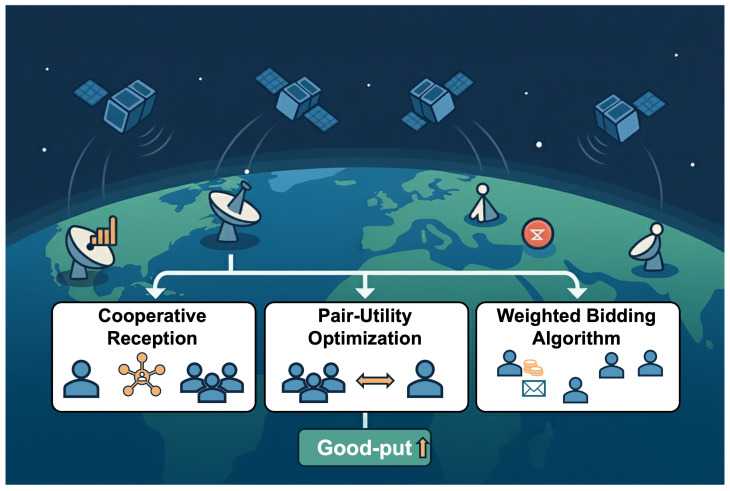
Overview of the three proposed algorithms for nanosatellite-to-ground communication: (1) Cooperative Reception (Shapley-value prioritization), (2) Pair-Utility Optimization (local pairwise refinement), and (3) Weighted Bidding (price-per-message allocation).

Unlike conventional greedy scheduling approaches that seek to maximize individual contact durations, these three methods explicitly prioritize global “good-put”. This shift in objective is designed to improve overall network throughput, fairness, and resilience (Figure 1). These algorithms are rigorously compared against current published methods to demonstrate their effectiveness in increasing data yield and efficiency within this challenging and dynamic operational environment.

The remainder of this paper is organized as follows. Section 2 reviews related work on satellite scheduling and distributed ground station networks. Section 3 describes the cooperative ground station architecture and formally defines the nanosatellite ground station scheduling problem. Section 4 introduces the proposed resource allocation framework and details the three algorithms for maximizing network-wide good-put: the Cooperative Reception Algorithm, the Pair Utility Optimization algorithm, and the Weighted bidding algorithm. Section 5 presents the simulation setup, parameter choices, and implementation details used to evaluate these methods, while Section 6 reports and compares the numerical results. Section 7 discusses the implications and limitations of the proposed approach and outlines possible extensions. Finally, Section 8 concludes the paper and highlights directions for future research.

## 2. Related Work

The literature on satellite communication scheduling is broadly characterized by two distinct streams: theoretical optimization focusing on large-scale missions, and practical implementation in open-source networks. Prior research has predominantly centered on Earth Observation Satellite (EOS) missions [4,5]. Foundational work in this domain, such as that by Wolfe and Sorensen [6], characterizes the problem as interval scheduling, often proving that finding optimal schedules under complex constraints is NP-hard. Consequently, rigorous formulations frequently employ Mixed Integer Linear Programming (MILP) or Constraint Satisfaction Problems (CSP) to strictly model visibility windows, energy constraints, and memory limitations. For example, Cho et al. [7] proposed a two-step binary linear programming formulation that provides high-quality solutions using standard MILP solvers. While MILP-based approaches provide exact or near-optimal solutions for centralized mission planning, they suffer from high computational complexity that scales poorly with network size. To address this, subsequent research has often pivoted toward heuristic and meta-heuristic approaches, such as genetic algorithms (GA) and particle swarm optimization (PSO) [8]. However, even these meta-heuristics typically assume a level of systemic control and global knowledge that is fundamentally absent in the low-cost, ad hoc nanosatellite environment. The computational overhead and reliance on centralized solvers render these classical approaches unsuited for distributed, decentralized networks where decisions must be made rapidly on low-power hardware [9,10].

Beyond purely greedy policies, several non-greedy scheduling and resource-allocation frameworks have been proposed for Earth observation and LEO satellite systems. Foundational work on EOS task scheduling describes the problem as a large-scale combinatorial optimization challenge with complex temporal and operational constraints, typically addressed by centralized planners with full knowledge of future passes and tasks [4,8]. A rich line of research then applies meta-heuristic and optimization-based methods, including genetic algorithms and related techniques, to improve schedule quality and handle agile constellations [7,9,11]. More recent work investigates centralized and distributed strategies for handover-aware task allocation in satellite constellations [12], as well as deep-learning-based multi-objective resource allocation for multi-band CubeSat communications [5]. In parallel, studies on efficient communication in networks of small LEO satellites and ground stations and on distributed and hybrid ground-station architectures [10,13] explore how to route traffic and share resources across multiple ground stations under operator control. These approaches generally focus on maximizing aggregate throughput, coverage, or latency-related objectives in tightly coordinated or operator-owned networks, and do not explicitly target the maximization of unique-message “good-put” in open, volunteer-operated networks such as SatNOGS and TinyGS [1,2]. In contrast, the algorithms introduced in this work are designed to operate under limited coordination in community ground-station deployments and to directly maximize the expected network-wide count of unique messages.

The operational landscape is being transformed by the rise of open-source ground station networks, which promote the democratization of space access. Projects like SatNOGS and TinyGS provide the infrastructure necessary for a global collective of operators to track independent LEO nanosatellites [1,2]. Given the increasing number of nanosatellites deployed by academic institutions for diverse research missions [14], these distributed networks are essential for handling the substantial growth in generated data. However, the operational scheduling within these networks often relies on simplistic, localized decision-making strategies, such as selecting the satellite with the best current signal-to-noise ratio, which, as discussed, results in significant message redundancy and network inefficiency.

This work critically adapts the core concepts of resource efficiency from the EOS literature to the realities of a distributed framework. Traditional scheduling aims to maximize total contact time or data volume; our focus, however, is on maximizing the network utility by ensuring the highest possible yield of unique messages. We model the system such that each nanosatellite transmits a single message at fixed intervals, and each ground station can only serve one frequency at a time. This formulation allows us to develop and evaluate resource allocation strategies that specifically overcome the limitations of centralized control and the inefficiencies of local-greedy decision-making, thereby providing a resilient and effective solution for maximizing the good-put in cooperative nanosatellite networks.

## 3. Distributed Ground Station Network

The SatNOGS project [1] represents significant work in the field of satellite distributed ground station networks. Ground stations are critical components of satellite communication systems responsible for tracking, receiving telemetry, monitoring, and facilitating command and control operations of satellites in orbit. The project’s focus on open source and modular design reflects a commitment to transparency, accessibility, and collaboration within the satellite communications community. With the growing number of LEO satellite launches worldwide, the demand for efficient, flexible, and cost-effective ground station solutions is increasing. SatNOGS addresses this challenge by offering a scalable and modular ground station concept that provides satellite operators with tools to track, identify, and communicate with their spacecraft. Using modern technologies such as low-cost software-defined radio and 3D printing, SatNOGS opens the door for individuals and organizations to set up ground stations that can actively contribute to the reception of satellite data.

**Figure 2:**
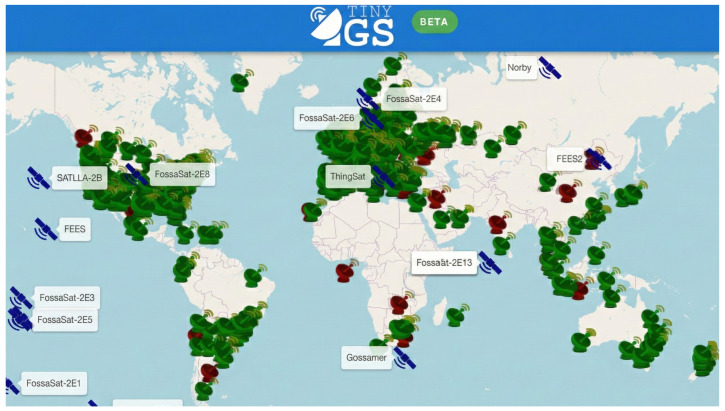
Global distribution of active ground stations in the TinyGS network.

Apart from the SatNOGS satellite ground station network, there is another project that offers comparable features. While SatNOGS relies on conventional modulation such as frequency shift keying (FSK) [1], TinyGS utilizes LoRa technology for satellite communication [3]. The open-source TinyGS project (Figure 2) democratizes nanosatellite tracking, enabling widespread contribution with minimal barriers. The network currently comprises over 1400 active stations and 4800 members globally [4]. Expanding this ground infrastructure offers a cost-effective method to enhance system performance compared to complex in-orbit updates.

The integration of LoRa technology in the TinyGS initiative offers significant advantages, enabling long-range and low-power communication between small satellites and ground stations. This integration enables the transmission and collection of data for various applications such as scientific research, remote sensing, and Earth observation. In addition, the low cost of building a TinyGS ground station makes it accessible to amateur satellite enthusiasts, students, and educational and research institutions with limited budgets.

LoRa, a proprietary modulation method, is commonly employed in IoT infrastructure solutions [15]. It operates on the chirp spread spectrum (CSS) principle, encoding data through wideband chirp signals with linear frequency changes. This technique is favored in IoT wireless networks due to its low power requirements and resilience against issues like multipath, fading, and the Doppler effect. LoRa significantly enhances receiver sensitivity by over 20 dB compared to traditional FSK, enabling extended communication ranges and reduced energy consumption, making it suitable not only for IoT but also for potential applications in nanosatellite communication systems, particularly CubeSats.

### 3.1. Motivation

Nanosatellite operators strive to maximize communication time and improve the reliability of their missions, which is critical to the success of various projects such as scientific research, environmental monitoring, and communication. To improve communication capabilities, some operators use a globally distributed network of ground stations, such as the SatNOGS and TinyGS networks. These networks are equipped with radio modules that enable the reception of nanosatellite data, and individuals can actively participate by registering their ground stations and scheduling reception. However, many existing resource management solutions for LEO networks often implement a greedy allocation strategy [7,12]. Each ground station independently selects the nanosatellite with the earliest available contact window. While this method maximizes the chance of successful communication at each station, it is widely recognized as a suboptimal approach for maximizing overall network performance [7].

**Figure 3:**
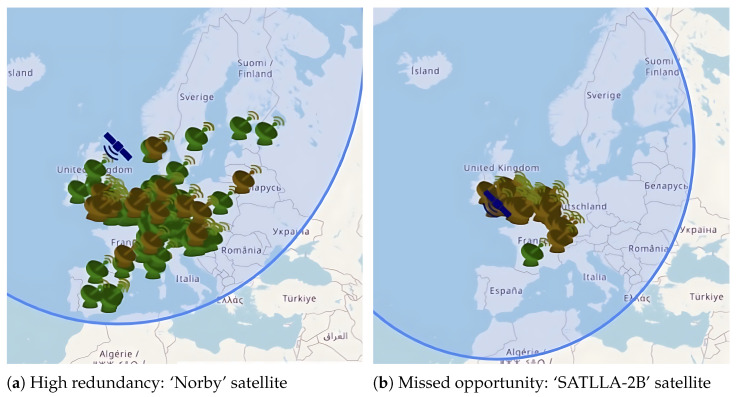
Impact of greedy scheduling on reception diversity. (**a**) Multiple stations simultaneously receive the strong ‘Norby’ signal (green icons). (**b**) Most stations ignore the weaker ‘SATLLA-2B’ signal, resulting in only a single successful reception.

In particular, the greedy algorithm leads to redundancy. As illustrated in Figure 3a, the high-power ‘Norby’ satellite is successfully received by numerous stations simultaneously. In contrast, Figure 3b shows the lower-power ‘SATLLA-2B’ satellite, where only a single station achieves successful reception while many others do not. This comparison highlights a critical flaw: ground stations independently prioritizing the strongest signal leads to high data redundancy, risking the complete loss of transmissions from weaker satellites.

This behavior results from a decision-making process that prioritizes the strongest signals while neglecting the broader network-wide goal of collecting unique messages. This technical inefficiency not only reduces data diversity, but also lowers the overall throughput of the network, as valuable downlink opportunities are wasted on duplicate messages instead of distributing reception more effectively. Optimization can not only improve data diversity, but also help mitigate radio interference—a growing concern in radio astronomy as satellite constellations inadvertently leak emissions into observational bands [16]. As the radio spectrum becomes increasingly crowded with terrestrial and satellite transmissions, a balanced approach to resource allocation is essential for maintaining effective and interference-minimized communications.

Addressing this imbalance is a critical technical challenge that motivates us to develop sophisticated strategies for efficient resource allocation in nanosatellite communications. Our approach goes beyond simple signal-based prioritization and introduces network-aware optimization techniques that dynamically adjust scheduling to maximize unique message reception across the network.

### 3.2. Problem Formulation

Effective management of satellite communication conflicts is a critical challenge. A nanosatellite typically passes over a given ground station in LEO about 2 to 3 times daily, with interactions lasting between 2 and 10 min. Consequently, determining which nanosatellite to track is essential.

**Figure 4:**
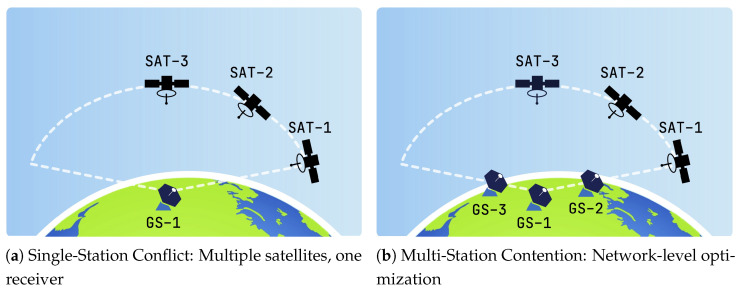
Visual representation of scheduling conflict scenarios. (**a**) A station faces a local exclusion constraint when multiple satellites pass simultaneously. (**b**) Dense orbital traffic creates complex combinatorial allocation problems across the network.

As illustrated in Figure 4a, a “Single-Station Conflict” arises when several nanosatellites pass over a ground station simultaneously; due to hardware constraints, the station can assigned to only one. The complexity scales in dense networks (Figure 4b), where multiple satellites have overlapping visibility windows with multiple ground stations. In this “Multi-Station Contention” scenario, efficient global allocation is critical to maximize data transmission. To formally address this, we establish the Nanosatellite Ground Station Scheduling Problem (NGSSP) as a Constraint Satisfaction Problem (CSP), defined over a fixed time horizon *T*.

#### 3.2.1. Sets, Parameters, and Variables

We define the following components for the NGSSP:

1. **Set of Nanosatellites:** N={1,2,…,n} is the set of *n* nanosatellites.
  **Assumption** **1.** *Each nanosatellite transmits a single message at fixed time intervals.*
  We denote the continuous sequence of unique messages transmitted by satellite *i* over the time horizon *T* as the set $M_{i}$.
2. **Set of Ground Stations/Receivers:** G={1,2,…,g} is the set of *g* receivers. We use the term ‘receiver’ as a satellite can also function as a relay.
3. **Visibility Parameter:** $a_{i,j}\left(t\right)\in \left\{0,1\right\}$ is a binary parameter indicating if satellite *i* is visible to receiver *j* at time *t*.
4. **Decision Variable:** $x_{i,j}\left(t\right)\in \left\{0,1\right\}$ is the binary decision variable, where $x_{i,j}\left(t\right)=1$ if receiver *j* is scheduled to receive from satellite *i* at time *t*, and 0 otherwise.

#### 3.2.2. Probability Model

The objective function defined in Equation (2) employs an expectation operator E[·] with respect to the inherent stochasticity of message decoding outcomes across the satellite–ground station links. For a specified time horizon *T*, orbital parameters (TLEs), and constellation setup, the calculated contact windows and pass geometries are treated as deterministic variables, derived from real-world data. The source of uncertainty stems from the successful reception of individual messages. For each nanosatellite–ground station pair (i,j) at time index *t*, we define $X_{i,j,t}\in \left\{0,1\right\}$ as a Bernoulli random variable indicating whether the message transmitted by satellite *i* is successfully decoded by ground station *j*. The probability of success is given by the decodability matrix:

(1) $$P\left(X_{i,j,t}=1\right)=P_{\mathrm{decode}}\left(i,j,t\right).$$

The values for $P_{\mathrm{decode}}\left(i,j,t\right)$ are derived directly from ground station dataset statistics, effectively capturing the aggregate effect of hardware performance and environmental factors. For tractability within our computational framework, we assume independence of decoding events across distinct message instances, conditioned on the corresponding $P_{\mathrm{decode}}$ entries. While operational networks may exhibit spatial correlation (e.g., 20–40%) in reception failures due to local environmental factors, explicitly modeling these joint probabilities is computationally prohibitive for edge devices. Therefore, the independence assumption implies that our simulation results represent a theoretical upper bound on network performance. Consequently, the expectation E[·] in Equation (2) is taken over the product probability space induced by the collection of these Bernoulli random variables $\left\{X_{i,j,t}\right\}$.

#### 3.2.3. Objective Function

The scheduling objective is to maximize the total number of unique messages received across the entire ground station network *G*. This goal is formalized as maximizing the expected (E) cardinality of the union of all successfully received unique message sets over the time horizon *T*:

(2) $$\mathrm{Maximize}:\;\;Z=E\left[\sum_{t\in T}\sum_{i\in N}\sum_{j\in G}x_{i,j}\left(t\right)\cdot X_{i,j,t}\cdot \lambda_{i}\left(t\right)\cdot \delta_{i,\mathrm{unique}}\left(t\right)\right]$$

where:

- $x_{i,j}\left(t\right)\in \left\{0,1\right\}$ is the scheduling decision variable that equals 1 if satellite *i* is allocated to transmit to ground station *j* at time *t*.
- $X_{i,j,t}\in \left\{0,1\right\}$ is the Bernoulli random variable for successful decoding of the message by station *j* at time *t* (defined in Section 3.2.2).
- $\lambda_{i}\left(t\right)$ is the data transmission rate of nanosatellite *i* at time *t*.
- m(i,t) is the unique identifier of the message transmitted by satellite *i* at time *t*.
- $M_{\mathrm{known}}\left(t\right)$ denotes the set of message IDs successfully decoded and registered by any ground station strictly before time *t*, maintained via a centralized MQTT broker (standard in TinyGS [2]) to prevent bottlenecks and resolve first-arrival order.
- $\delta_{i,\mathrm{unique}}\left(t\right)$ is a binary factor that equals 1 only if the message being received is unique to the network at time *t*. This is mathematically defined as:
  (3) $$\delta_{i,\mathrm{unique}}\left(t\right)=I\left(m\left(i,t\right)\notin M_{\mathrm{known}}\left(t\right)\right)$$
  where I(·) is the indicator function. Successful decoding and scheduling are captured separately by $x_{i,j}\left(t\right)$ and $X_{i,j,t}$. This factor explicitly captures the unique message requirement.
  **Assumption** **2.** *A message contributes to the network utility (good-put) only if it is unique; redundant receptions of the same message ID by multiple stations at the same time or strictly after a previous reception are considered to have zero marginal utility.*

In a more general version, positive weights can be assigned to messages to maximize the total weighted reward.

#### 3.2.4. Constraints

The scheduling must adhere to the physical and operational constraints of the distributed network:

1. Exclusive Receiver Use (Single Assignment): A receiver can only serve (track and decode) one nanosatellite at any given time *t*.
  (4) $$\sum_{i\in N}x_{i,j}\left(t\right)\leq 1\;\forall j\in G,t\in T$$
2. Visibility Constraint: A receiver *j* can only be assigned to satellite *i* if *i* is within *j*’s visibility window.
  (5) $$x_{i,j}\left(t\right)\leq a_{i,j}\left(t\right)\;\forall i\in N,j\in G,t\in T$$
3. Binary Constraint: The decision variable must be binary.
  (6) $$x_{i,j}\left(t\right)\in \left\{0,1\right\}\;\forall i\in N,j\in G,t\in T$$

**Remark 1** (**Computational Complexity**)**.**
*The NGSSP objective of maximizing unique message reception under time-varying visibility and assignment constraints forms a combinatorial selection problem closely related to the classical Maximum Coverage Problem, which is known to be NP-hard. Furthermore, similar visibility-constrained scheduling formulations in the Earth Observation literature have been shown to be NP-hard [6]. Consequently, computing a global optimum becomes intractable for large-scale constellations, motivating the use of the efficient polynomial-time heuristics proposed in Section 4.*

## 4. Proposed Method

We introduce new algorithms for ground station allocation that explicitly account for factors such as the reception probability of each nanosatellite at a given ground station, the distance between satellites and stations, and their respective geographic locations. Our approach seeks not only to maximize the overall efficiency of data transmission but also to guarantee equitable communication opportunities for all nanosatellites. Specifically, our primary objective is to maximize the number of unique messages (good-put) successfully delivered across the distributed network, while ensuring fairness among satellites. In contrast to conventional nanosatellite ground station networks, which typically rely on simple greedy algorithms that prioritize the nearest nanosatellite for communication, the effectiveness of such approaches in scenarios requiring global non-redundancy remains uncertain. To provide a rigorous evaluation, we adopt the standard greedy algorithm as a baseline and benchmark it against our more advanced utility-based heuristics. While probabilistic greedy variants (e.g., selecting the satellite with the highest $P_{rx}$) were considered, they exhibit the same structural flaw identified in Section 3.1: a tendency for multiple stations to redundantly select the strongest signal, leaving weaker signals unserved. Consequently, the standard greedy baseline effectively represents the performance ceiling of local strategies that lack coordination.

In parallel, prior work has explored non-greedy optimization strategies for satellite scheduling and resource allocation, including centralized EOS formulations, meta-heuristic and optimization-based methods, and constellation-level task allocation schemes [4,7,8,9,11,12], as well as deep-learning-based resource allocation for multi-band CubeSat networks and distributed or hybrid ground-station architectures [5,10,13]. However, these approaches typically assume operator-controlled space and ground segments with strong coordination and focus on maximizing aggregate throughput, coverage, or latency. In contrast, our evaluation concentrates on greedy and probability-aware greedy baselines that are directly implementable in open volunteer networks, while our unique-message good-put objective could in principle be embedded as a reward or constraint in the above centralized frameworks.

While heuristic in nature, our algorithms are grounded in a formal theoretical framework and accompanied by a comprehensive complexity analysis. Moreover, they embody a specialized adaptation of established techniques, tailored to address the unique challenges of non-redundant nanosatellite communication. The innovation of the proposed methods does not stem from the development of entirely new theoretical frameworks, but rather from the specialized adaptation and computational refinement of established techniques to address the distinctive challenges of decentralized scheduling in conjunction with the unique-message objective inherent to LEO nanosatellite networks. Each algorithm is supported by a precise mathematical formulation and a formal analysis of computational complexity. Although heuristic in nature and not guaranteed to achieve global optimality, our approach leverages a rigorous, tailored formulation of utility and reward functions—explicitly designed to incorporate global message history within a decentralized setting—alongside extensive simulation studies to thoroughly assess performance. This combination highlights that the principal complexity lies in the customized formulation itself, which both confronts the key challenges of decentralized resource allocation in ground station networks and establishes a robust theoretical foundation for practical deployment.

### 4.1. Cooperative Reception Algorithm

To optimize the efficiency of nanosatellite communications, this algorithm applies cooperative game theory principles to quantify the contribution of each ground station within the network. Based on these utility assessments, Algorithm 1 then selects the upcoming nanosatellite pass that is most likely to maximize collective message reception. This evaluation takes into account characteristics such as reception quality, geographic location and satellite transmission patterns to maximize the collective reception of unique messages across the network. Let *N* be the set of all participating ground stations, and let *M* be the set of all possible messages transmitted by a nanosatellite during a certain period of time (e.g., a pass). Let $M_{i}$ be the set of messages uniquely received by ground station i∈N, and M(S) the set of unique messages received by any station in a coalition S⊆N. The utility function for a coalition *S* is defined by the number of unique messages it collectively receives:

(7) v(S)=|M(S)|

where |M(S)| represents the cardinality of the set of unique messages successfully received by at least one station in *S*. This metric treats all unique messages as equally valuable, a simplification suitable for demonstrating the cooperative framework’s allocation mechanism. The marginal contribution of a station *i* to a coalition *S* is then:

(8) v(S∪{i})−v(S)=|M(S∪{i})|−|M(S)|.

This measures the additional unique messages captured only when station *i* joins coalition *S*. A station primarily receiving redundant messages (already captured by others in *S*) will have a low marginal contribution. To fairly distribute credit for received messages among the cooperating ground stations, we use the Shapley value from cooperative game theory [17]. The Shapley value for a station *i*, denoted as $\phi_{i}$, quantifies its average marginal contribution to all possible coalitions it could join within the network *N*:

(9) $$\begin{array}{ccc} \phi_{i}= & \sum_{S\subseteq N\setminus \{i\}}\frac{|S|!(|N|-|S|-1)!}{|N|!}\; & \left[v(S\cup \{i\})-v(S)\right] \end{array}$$

The Shapley value ensures a fair and stable distribution by systematically averaging a station’s contribution across all cooperation scenarios [17]. It rewards stations that significantly improve the network’s unique message capture, as opposed to simpler methods that may only reward high volume or treat all stations equally. While the Shapley value $\phi_{i}$ evaluates the importance of the individual stations, Algorithm 1 aims to select the satellite pass that maximizes the collective network performance. By the efficiency property of cooperative game theory, the sum of all individual Shapley values equals the utility of the grand coalition, v(N). Consequently, the function ComputeCooperativeUtility calculates this total network utility—representing the aggregate expected number of unique messages captured by the entire network—to rank and select the optimal satellite pass. This approach utilizes the marginal contribution logic underlying the Shapley value to efficiently estimate the collective value generated by the network *N*, implicitly rewarding stations that improve overall coverage by capturing transmissions missed by others.

Analyzing historical message reception data allows the algorithm (via its utility calculation) to identify stations that are critical for capturing transmissions missed by others. If a satellite’s signals are rarely received, the stations that do capture them contribute significantly to v(N), increasing the calculated utility for passes of that satellite involving those stations. Conversely, for easily received satellites, the marginal contribution of any single station is less critical to the total v(N). This ensures that the system implicitly rewards stations that improve the overall coverage.
**Algorithm 1** Cooperative Reception Algorithm**Input:** TLE set (Two-Line Element data), Ground stations set *N*, location (observer coordinates)**Output:** sat_id of the best upcoming pass based on predicted network utility  1:passes ← UpcomingSatellitePasses(*TLE_s_*, *location*)  2:**if** passes is empty **then return** No passes available  3:**end if**  4:**if** size(passes) = 1 **then return** passes[0].sat_id  5:**end if**  6:max_expected_utility ← −∞  7:best_pass ← NULL  8:**for** each pass in passes **do**  9:    pass_utility ← ComputeCooperativeUtility(*pass*, *N*)10:    **if** pass_utility > max_expected_utility **then**11:        max_expected_utility ← pass_utility12:        best_pass ← pass13:    **end if**14:**end for**15:**return** best_pass.sat_id

The exact calculation of Shapley values has an exponential complexity of $O(2^{\left|N\right|})$, which is impractical for real-time decision-making. To address this, ComputeCooperativeUtility employs the standard Monte Carlo estimator described by Mitchell et al. [17], approximating the value by sampling random permutations of ground stations and averaging the marginal contribution of each station across these samples. For a future pass, we define the characteristic function v(S) as the expected utility of the coalition *S*, quantified as the probability that at least one station in *S* successfully decodes the message:

(10) $$v\left(S\right)=1-\prod_{j\in S}\left(1-P_{rx}\left(j\right)\right)$$

where $P_{rx}\left(j\right)$ is the estimated reception probability for station *j* (derived directly from the ground station dataset statistics and accessible via the coordination service mentioned in Section 3.2.3). Specifically, the ComputeCooperativeUtility function generates *k* random permutations of the network. For a given permutation σ and station *i*, let Pre(σ,i) be the set of stations preceding *i*. The marginal contribution of station *i* is the increase in reception probability provided by adding *i* to its predecessors. This simplifies to:

(11) $$\Delta_{i}\left(\sigma \right)=v\left(Pre\left(\sigma ,i\right)\cup i\right)-v\left(Pre\left(\sigma ,i\right)\right)=P_{rx}\left(i\right)\cdot \prod_{j\in Pre(\sigma ,i)}\left(1-P_{rx}\left(j\right)\right)$$

The approximate Shapley value $\phi_{i}$ is the average of these marginal contributions over the *k* samples. This formulation explicitly rewards stations that provide unique coverage—those that are likely to receive the message when others (preceding them in the permutation) fail.

**Proposition 1** (Convergence of Monte Carlo Shapley Estimator)**.** *The Monte Carlo Shapley estimates based on K random permutations are unbiased estimates of the corresponding true Shapley values as established in Proposition 1 of [17], and their typical estimation error decreases at the standard Monte Carlo rate (on the order of $1/\sqrt{K}$). Consequently, for any fixed finite set of candidate passes, the probability that the pass selected using these estimates has substantially lower true cooperative utility than the optimal pass tends to zero as K increases. In particular, when utilities are bounded and non-negative, the expected approximation ratio between the utility achieved by our method and the optimal game-theoretic utility converges to 1 as K grows.*

### 4.2. Pair Utility Optimization Algorithm

Building on the foundations established in Algorithm 1, Algorithm 2 introduces a refined local pairwise comparison method between ground stations to optimize nanosatellite pass assignments. In this approach, the utility of each ground station for a given nanosatellite pass event is evaluated by comparing the probability of successfully receiving a message with that of neighboring stations. By quantifying the differences in reception probabilities, the algorithm mitigates selection bias and preferentially assigns nanosatellite passes to stations that are best positioned relative to their local neighbors. In addition, the algorithm continuously adapts to network conditions in real time by updating reception probabilities and other relevant parameters, ensuring robust and efficient allocation of nanosatellite observation opportunities.

Let *i* denote the ground station executing the algorithm, identified by its specific gs_id_*i*_ and geographic ${\mathrm{location}}_{i}$.

Let N(i) denote the set of neighboring ground stations to station *i*. To ensure reproducibility, we strictly define the neighborhood based on geographic proximity. A ground station *k* is a member of the set N(i) if and only if the geodesic distance between *i* and *k* is less than or equal to a fixed radius $R_{neighbor}$.

$$N\left(i\right)=\{k\in G|\mathrm{Distance}\left(i,k\right)\leq R_{neighbor},k\neq i\}$$

In our simulations, we set $R_{neighbor}=50$ km, corresponding to the average station spacing observed in the network topology (Table 1). This definition ensures that pairwise comparisons are restricted to stations that are statistically likely to compete for the same satellite transmission windows due to overlapping visibility cones.

Let *p* denote a particular nanosatellite pass event, and let S(p) be the nanosatellite associated with the pass event *p*. Let $P_{rx}\left(s,p\right)$ denote the estimated probability that the station *s* successfully receives a message from the nanosatellite S(p) during the pass event *p*.

The local utility U(i,p) for the station *i* during the pass event *p* is defined as the joint probability that the station *i* receives the message and that none of its neighboring stations k∈N(i) does so, assuming independence between the reception events:

(12) $$U\left(i,p\right)=P_{rx}\left(i,p\right)\times \prod_{k\in N(i)}\left(1-P_{rx}\left(k,p\right)\right)$$

This utility formulation intentionally assumes maximal contention to mitigate redundancy without requiring real-time schedule exchange. We note that this formulation acts as a sparsity filter: as the neighborhood density |N(i)| increases, the utility U(i,p) decays exponentially. This behavior effectively penalizes stations in dense clusters, where coverage is likely redundant. By treating all visible neighbors as active competitors, the algorithm quantifies the local value of a pass for station *i*, giving preference to opportunities where station *i* has a comparative advantage. This conservative approach ensures that even if neighbors are busy with other tasks, station *i* prioritizes signals that the neighborhood is statistically less likely to capture, thereby maximizing the expected number of unique messages across the distributed network.
**Algorithm 2** Pair Utility Optimization**Input:** TLE set (Two-Line Element data), ID (observer ID), location (observer coordinates)**Output:** sat_id of the best upcoming pass based on the local utility heuristic U(i,p) defined in Equation (12)  1:passes ← UpcomingSatellitePasses(*TLE_s_*, *location*)  2:**if** passes is empty **then return** No passes available  3:**end if**  4:**if** size(passes) = 1 **then return** passes[0].sat_id  5:**end if**  6:max_utility_found ← −∞  7:best_pass ← NULL  8:neighbors ← GetNeighbors(ID)  9:**for all** pass in passes **do**10:    prob_rx ← CalculateRxProb(ID, pass)11:    utility_for_pass ← prob_rx12:    **for all** neighbor in neighbors **do**13:        prob_neighbor_rx ← CalculateRxProb(neighbor, pass)14:        utility_for_pass ← utility_for_pass × (1 − prob_neighbor_rx)15:    **end for**16:    **if** utility_for_pass > max_utility_found **then**17:        max_utility_found ← utility_for_pass18:        best_pass ← pass19:    **end if**20:**end for**21:**return** best_pass.sat_id

**Example 1: Pair Utility Optimization.**
Consider neighboring ground stations ${\mathrm{GS}}_{1}$ and ${\mathrm{GS}}_{2}$ which have upcoming observation opportunities for Satellite A and Satellite B. Satellite A is a high-power nanosatellite whose pass is favorable for both stations; ${\mathrm{GS}}_{1}$ has $P_{rx}=0.8$ and ${\mathrm{GS}}_{2}$ has $P_{rx}=0.7$. Satellite B is a low-power nanosatellite. It passes over both ${\mathrm{GS}}_{1}$ and ${\mathrm{GS}}_{2}$, but due to its limited power the probability of successful reception is only moderate at ${\mathrm{GS}}_{1}$$P_{rx}=0.6$ and very low at ${\mathrm{GS}}_{2}$$P_{rx}=0.1$. A greedy algorithm would have both stations select Satellite A, resulting in message redundancy. The estimated reception probabilities are given by
  $$\begin{array}{cc} P_{rx}\left({\mathrm{GS}}_{1},A\right)=0.8, & P_{rx}\left({\mathrm{GS}}_{1},B\right)=0.6,\\ P_{rx}\left({\mathrm{GS}}_{2},A\right)=0.7, & P_{rx}\left({\mathrm{GS}}_{2},B\right)=0.1. \end{array}$$
Each station computes its local utility U(i,p) for each satellite p∈{A,B} using Equation (12). For ${\mathrm{GS}}_{1}$:
  $$\begin{array}{cc} U({\mathrm{GS}}_{1},A) & =0.8\cdot (1-0.7)=0.24,\\ U({\mathrm{GS}}_{1},B) & =0.6\cdot (1-0.1)=0.54. \end{array}$$
Since 0.54>0.24, station ${\mathrm{GS}}_{1}$ selects Satellite B.

For ${\mathrm{GS}}_{2}$:

$$\begin{array}{cc} U({\mathrm{GS}}_{2},A) & =0.7\cdot (1-0.8)=0.14,\\ U({\mathrm{GS}}_{2},B) & =0.1\cdot (1-0.6)=0.04. \end{array}$$

Thus, station ${\mathrm{GS}}_{2}$ selects Satellite A.

This example shows that the local utility heuristic defined in Equation (12) successfully guides individual ground stations to promote distinct nanosatellite selection. By systematically avoiding redundant tracking, which would otherwise cause both stations to choose Satellite A (the strongest signal), the algorithm ensures that the unique message from Satellite B is also captured. This strategic diversification ultimately maximizes the expected reward, defined as the total number of unique messages received across the network.

### 4.3. Weighted Algorithm

The third algorithm, Algorithm 3, addresses the unique operating environment of networks such as TinyGS and SatNOGS, whose operators receive no direct financial compensation. Following the reward system of the Helium network [18], we build in a bidding mechanism that assigns a price per message to each satellite operator. The expected reward for each ground station is calculated as

(13) $$r_{gs}=\frac{pb\left(gs,m\right)\cdot r_{m}}{k},$$

where pb(gs,m) is the probability that the ground station gs receives the message *m*, $r_{m}$ is the reward per message, and *k* is the number of ground stations competing for this message.

To implement this, the Weight(sat_id) function assigns a scalar value $r_{m}$ to each satellite, representing the operator’s assigned priority or ‘bid’ for data retrieval. In a practical deployment, this scalar could be dynamically adjusted by operators through a bidding dashboard to reflect immediate mission needs—such as increasing the ‘price’ during critical launch and early orbit phases —or established through a fixed service-level agreement. A higher weight indicates critical data (e.g., real-time telemetry) or a higher willingness to utilize network resources.

The function EstimateCompetingStations calculates $k_{est}$, the expected number of other ground stations that will have visibility of the satellite during the target pass. This is determined deterministically using the TLE sets and the known coordinates of the network’s ground stations. By identifying all stations j∈G where the visibility parameter $a_{sat,j}\left(t\right)=1$ for the given time window, the algorithm predicts the ‘dilution’ of the reward:

(14) $$k_{est}=\sum_{j\in G}a_{sat,j}\left(t\right)$$

This mechanism naturally incentivizes ground stations to select passes with fewer competitors, thereby broadening the network’s effective coverage. Although utilizing historical reception probabilities would refine the effective competition count, we retain this binary formulation to ensure a conservative lower-bound reward estimate and minimize computational overhead. Future iterations may incorporate station reliability scores to further fine-tune this scarcity factor.
**Algorithm 3** Weighted Algorithm**Input:** TLE set, IS (observer ID), location (observer coordinates), Weight(), RxProbability()**Output:** sat_id of the best upcoming pass based on predicted network utility  1:passes ← UpcomingSatellitePasses(*TLE_s_*, *location*)  2:**if** passes is empty **then return** No passes available  3:**end if**  4:**if** size(passes) = 1 **then return** passes[0].sat_id  5:**end if**  6:max_expected_reward ← −∞  7:best_pass ← NULL  8:**for all** pass **in** passes **do**  9:    sat_id ← pass.sat_id10:    weight ← Weight(sat_id)11:    rx_prob ← RxProbability(sat_id)12:    $k_{est}$← EstimateCompetingStations(sat_id, location)13:    expected_reward ← (weight * rx_prob) / $k_{est}$14:    **if** expected_reward > max_expected_reward **then**15:        max_expected_reward ← expected_reward16:        best_pass ← pass17:    **end if**18:**end for**19:**return** best_pass.sat_id

### 4.4. Computational Complexity Analysis

To assess feasibility for real-time decision-making, we analyze the complexity relative to the number of ground stations ($N_{GS}$), trackable satellites ($N_{SAT}$), and Monte Carlo samples (*K*).

- **Cooperative Reception (Algorithm 1):** The exact Shapley value requires $O(2^{N_{GS}})$, which is computationally prohibitive. Our Monte Carlo approximation reduces the time complexity to $O(K\cdot N_{SAT}\cdot N_{vis})$. We note that while $N_{GS}$ denotes the total network size, practical iterations are restricted to the subset of stations $N_{vis}\ll N_{GS}$ that are visible to the satellite. Space complexity is $O(N_{SAT}\cdot N_{GS})$ for the probability matrix.
- **Pair Utility Optimization (Algorithm 2):** This algorithm operates on local neighborhoods. Let $D_{max}$ be the maximum degree of the network graph. The time complexity is $O(N_{SAT}\cdot D_{max})$. Since neighbor lists are cached and $D_{max}\ll N_{GS}$ in distributed networks, this is highly efficient, typically executing in milliseconds. Space complexity is $O(N_{GS}+E)$ for the adjacency list.
- **Weighted Algorithm (Algorithm 3):** Calculating the competition factor $k_{est}$ requires checking global visibility for all stations. The time complexity is $O(N_{SAT}\cdot N_{GS})$, with space complexity dominated by the visibility data $O(N_{SAT}\cdot N_{GS})$.

All three algorithms exhibit polynomial time complexity, with orbital propagation and visibility pre-calculated and cached. Given typical network sizes ($N_{GS}\approx 2000$) and sampling rates (K≈100), these methods are computationally lightweight and well-suited for execution on standard edge hardware (e.g., Raspberry Pi) or central servers with negligible latency.

## 5. Simulation

**Figure 5:**
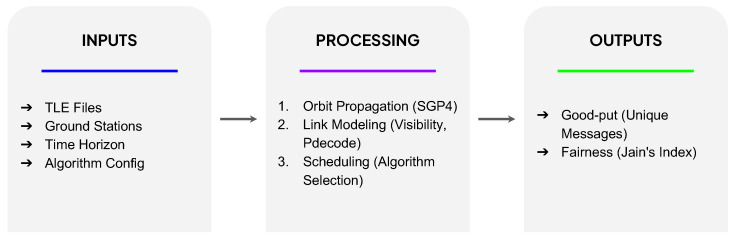
Simulation framework architecture: TLE and ground station inputs are processed via SGP4 and link modeling to generate probability matrices for scheduling algorithm evaluation.

In order to evaluate and optimize the interaction between nanosatellites and ground stations, we developed a comprehensive simulation framework that models the dynamics of nanosatellite communication under real conditions. The system architecture, illustrated in Figure 5, processes orbital elements (TLEs) and station parameters to generate contact windows and probability matrices for the scheduling algorithms.

**Figure 6:**
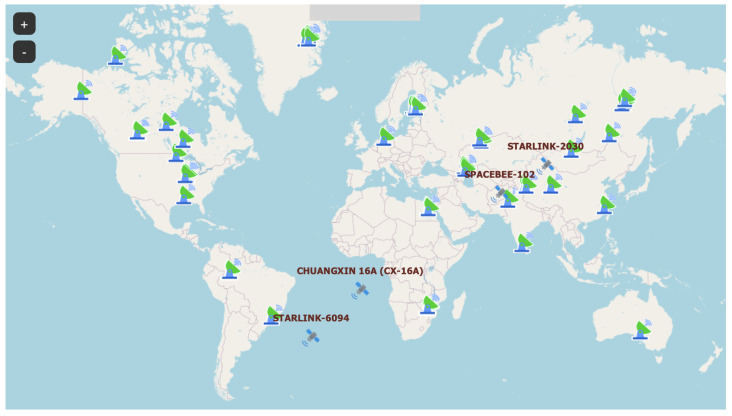
Simulation environment topology consisting of 51 nanosatellites and 92 ground stations.

Within this framework, we established the network topology shown in Figure 6, consisting of 51 active nanosatellites and 92 ground stations. To ensure operational realism, the satellite orbits are derived from real-world TLE data [19] rather than a synthetic constellation model. This heterogeneous mix spans inclinations from $53^{\circ }$ to $98^{\circ }$ (Sun-Synchronous) and altitudes of 400–550 km, resulting in a global average revisit time of approximately 45 min. While operational networks exhibit dynamic behavior with stations frequently joining or leaving, this simulation assumes a static topology to isolate scheduling performance from stochastic failures. However, it is critical to note that the proposed algorithms utilize a stateless, just-in-time execution model. Because utility is recalculated in real-time before each pass, node unavailability (churn) is computationally treated as equivalent to visibility loss. Consequently, the system inherently adapts to topological volatility without the fragility associated with pre-computed schedules.

In sparse network scenarios where ground station visibility windows rarely overlap, simple greedy heuristics are often sufficient. However, the chosen ratio of satellites to ground stations creates a high-contention environment where multiple satellites frequently compete for limited ground station slots, and conversely, single stations are subject to simultaneous pass requests. This density effectively stress-tests the proposed algorithms’ ability to resolve conflicts and mitigate redundancy, providing a valid representation of the scheduling bottlenecks observed in dense regional clusters of networks like SatNOGS or TinyGS.

The locations of the ground stations are derived from publicly available layout data on the TinyGS website. The orbital parameters of the satellites are determined using TLE data from CelesTrak website [19], which is retrieved based on North American Aerospace Defense Command (NORAD) identification. Their sky positions are calculated using the Python package Skyfield (v1.47) [20], which implements the SGP4 propagation model to ensure high-fidelity orbital prediction. This setup results in dynamic contact windows ranging from 8 to 14 min per pass, consistent with the physics of LEO nanosatellites at 500 km altitude.

The TLE data serves as a standardized representation of the characteristics of an object in orbit, including unique identifiers and parameters such as latitude and longitude [13]. Global Positioning System (GPS) data is used to enable precise distance calculations between satellites and ground stations. The simulator calculates the time windows for communication between nanosatellite and ground station by integrating the nanosatellite position data with the geographical coordinates of each station.

To provide a precise overview of the simulation environment and address the heterogeneity of the system parameters, we present the configuration data in two separate tables. Table 1 details the general simulation control settings and satellite configurations.

The specific characteristics of the distributed ground station network are summarized in Table 2.

Each nanosatellite transmits messages at fixed intervals, initially set to 60 s, based on standard nanosatellite communication practices. This value serves as a baseline, but the simulator allows adjustments to investigate the effects of varying transmission frequencies. Each transmitted message carries a unique identifier and the main objective is to track the success of the messages successfully received by ground stations. The probability of successful message decoding is represented using a decodability probability matrix, which quantifies the likelihood of successful reception for each nanosatellite-ground station pair. To accurately model the heterogeneous nature of volunteer networks—where specific hardware parameters (e.g., antenna gain patterns, system noise temperature) are often unknown—we utilize an empirical approach rather than a theoretical link budget model. The entries for $P_{decode}$ are derived directly from historical TinyGS reception statistics relative to satellite elevation and distance. Consequently, physical layer factors such as SNR thresholds, free-space path loss, and atmospheric attenuation are implicitly captured within the aggregated historical performance data, ensuring the simulation reflects real-world operational conditions. To maintain the fidelity of these probability estimates in a long-term deployment, we recommend a quarterly recalibration of the $P_{decode}$ matrix to account for hardware upgrades and orbital evolution. However, it is important to note that while this empirical approach captures average link performance, specific intra-pass dynamics such as rapid Doppler shift compensation and small-scale multipath fading are not explicitly modeled.

For verification and reproducibility, the simulator runs with fixed random seeds and consistent date ranges across all experiments. To ensure operational realism, the simulation mirrors the live network topology using real-world TLE data and ground station coordinates. Furthermore, we verified that the baseline throughput aligns with typical daily message volumes observed in the operational TinyGS network.

## 6. Algorithms Evaluation

To rigorously assess the performance of the proposed methods, we benchmark them against a standard Greedy Earliest-Contact Scheduling algorithm. Although complex systems may prioritize passes based on signal-to-noise ratios or elevation angles, distributed ground stations often use a temporal maximization strategy to minimize idle time. In this baseline approach, the ground station identifies all upcoming satellite passes within a prediction horizon (e.g., 24 h) and selects the satellite with the earliest AOS time.

This heuristic interprets “proximity” in a temporal rather than spatial sense, ensuring that each ground station attempts to initiate contact with the next satellite that becomes available, irrespective of pass duration or signal quality. The formal procedure for this baseline is detailed in Algorithm 4.
**Algorithm 4** Baseline Greedy Scheduler (Earliest Contact)**Input:** Set of Nanosatellites *S*, Ground Station GS, Current Time $T_{now}$, Prediction Horizon *H***Output:** next_pass object selected for tracking  1:overpasses ← GetUpcomingPasses$\left(S,GS,T_{now},H\right)$  2:time_to_next_pass ← ∞  3:next_pass ← NULL  4:**for all** pass **in** overpasses **do**  5:    $t_{start}$← GetStartTime(pass)  6:    time_difference ←$t_{start}-T_{now}$  7:    **if** time_difference < time_to_next_pass **then**  8:        time_to_next_pass ← time_difference  9:        next_pass ← pass10:    **end if**11:**end for**12:**return** next_pass

To quantify the efficiency of the proposed algorithms, we establish a theoretical upper bound for the network’s throughput. We calculate an idealized capacity of approximately 29,000 unique messages for the entire network over the 24-h simulation period. This value is derived by summing the expected message yield of all potential nanosatellite-to-ground contact windows—weighted by their respective reception probabilities ($P_{\mathrm{decode}}$)—under the relaxed assumption that no visibility conflicts exist and all potential links can be utilized simultaneously. This upper bound serves as a reference point to evaluate how closely the practical scheduling algorithms approach the theoretical limit of the physical infrastructure.

**Figure 7:**
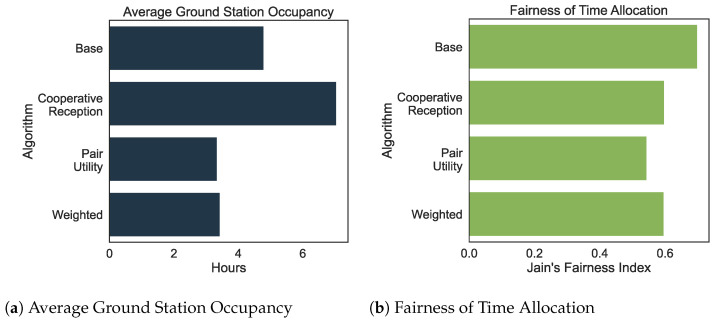
Performance metrics for different satellite selection algorithms. (**a**) The average allocation of listening time per satellite over a 24-h period. (**b**) The corresponding Jain’s Fairness Index for the time allocation.

Simulation parameters, including satellite pass frequency, ground station density, and communication window duration, were calibrated against operational data from SatNOGS and TinyGS to reflect realistic network conditions. The fairness of the nanosatellite listening time distribution is quantified using Jain’s Fairness Index, defined as:

(15) $$J=\frac{{\left(\sum_{i=1}^{n}x_{i}\right)}^{2}}{n\cdot \sum_{i=1}^{n}x_{i}^{2}},$$

where $x_{i}$ denotes the total listening time allocated to satellite *i* and *n* is the total number of satellites. A value of J=1 corresponds to perfect fairness, while values below 1 indicate increasing disparity. This metric provides insight into how equally listening time is distributed among the satellites, which is critical when balancing fairness with overall system throughput. Figure 7a compares the average daily listening times allocated to satellites, while Figure 7b illustrates the corresponding fairness indices for each algorithm. The baseline algorithm, which employs a greedy heuristic, achieves the highest fairness index of 0.6993 with an average listening time of 4:47:30 per day. Although this balanced allocation reflects a high degree of fairness, it comes at the cost of limiting the overall throughput.

**Figure 8:**
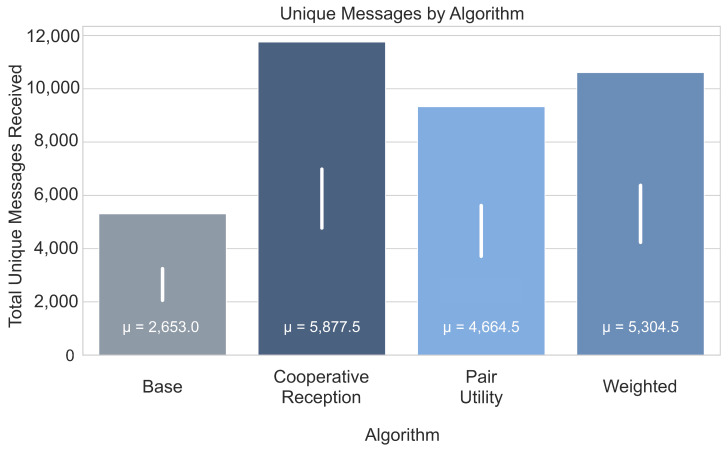
Total unique messages received by the network under different satellite selection algorithms during a 24-h simulation. The value μ inside each bar denotes the mean number of unique messages, and the white vertical line shows the standard deviation.

In contrast, the proposed Cooperative Reception algorithm (Figure 8) significantly increases throughput, nearly doubling the number of unique messages received compared to the baseline. This improvement is achieved by proactively prioritizing satellite passes based on the marginal contribution of ground stations to the global message set. However, this optimization introduces a quantifiable trade-off in service equity. By favoring satellites that contribute unique data—often those with weaker links or fewer redundant observers—the algorithm deviates from a uniform distribution of contact time.

Consequently, the fairness metric declines relative to the greedy baseline. While the baseline achieves a Jain’s Fairness Index of 0.6993, the Cooperative Reception, Pair Utility Optimization, and Weighted algorithms record indices of 0.5982, 0.5446, and 0.5966, respectively. This represents a reduction in fairness of approximately 14% to 22%. However, this shift is an expected and desirable trade-off given the change in objective. To maximize global good-put (unique message diversity), the network must allocate disproportionate resources to “harder-to-receive” satellites, prioritizing efficiency over equitable contact time. Thus, the reduction in strict fairness is necessary to minimize redundancy and maximize network utility.

Figure 7 and Figure 8 provide a comprehensive overview of the key performance indicators recorded during a 24-h simulation. The simulation utilizes deterministic real-world TLE data, ensuring that orbital geometry and contact windows are fixed and reproducible. This stability eliminates the need for transient warm-up periods typically required in stochastic models. As illustrated by the error bars in Figure 8, the variance driven by the probabilistic link layer is minimal relative to the performance gains, confirming that the superiority of the proposed algorithms is robust and statistically distinct.

## 7. Discussion

This paper addresses nanosatellite data downlink challenges through a resource allocation problem, where the optimization framework considers nanosatellite transmission power, satellite-ground station distances, and RF interference to enhance network performance. The study evaluates three algorithms for nanosatellite selection, comparing their effectiveness in maximizing unique message reception and ensuring fairness in resource distribution. Simulation results indicate that, compared to the commonly used greedy baseline algorithm, the proposed methods nearly double the number of unique messages successfully received across the network. This improvement is achieved by optimizing the satellite selection based on expected message reception instead of relying only on proximity. However, this gain comes at the cost of a slight reduction in fairness as measured by the Jain’s Fairness Index.

The proposed algorithms are not only numerically robust but also theoretically advantageous. They provide scalability for numerous satellites and ground stations, resilience to topology changes, and real-time computational efficiency. Overall, these algorithms represent a promising solution for resource allocation in nanosatellite networks. This has implications for improved communication performance and applications such as satellite-based IoT services and remote sensing.

Beyond theoretical throughput gains, the practical viability of these algorithms depends on their execution within the tight processing windows of low-cost ground stations. As detailed in the complexity analysis (Section 4.4), the proposed heuristics operate in polynomial time. This efficiency is critical for decentralized networks like TinyGS, where stations often rely on resource-constrained microcontrollers (e.g., ESP32) rather than full single-board computers. In our simulations, the decision logic for all three algorithms executes in milliseconds—orders of magnitude faster than the standard 60-s inter-message transmission interval. This ensures that scheduling decisions can be performed in real-time without inducing latency that would result in missed transmission windows. Furthermore, the low computational overhead minimizes energy consumption, a vital consideration for off-grid, solar-powered ground stations common in the amateur radio community.

The proposed algorithms can be extended in several directions to bridge the gap between simulation and operational deployment. First, while we compared our approach against standard greedy baselines, future work will involve quantifying the suboptimality gap against formal MILP formulations on smaller network instances. This will provide stricter theoretical performance bounds.

Second, we aim to investigate the algorithms’ resilience under adversarial conditions, specifically severe link degradation, major ground station outages, and Byzantine behaviors (e.g., false reception reporting). “Stress testing” the system under these volatile conditions is essential to ensure reliability before large-scale adoption.

Finally, validating the algorithms with real-world data and hardware represents a critical milestone. While the probability matrices used in this study were derived from aggregated historical reception statistics to approximate typical link performance, we acknowledge that this dataset represents a static snapshot and may not fully capture the temporal variability or long-term representativeness of dynamic network conditions. To address this, we plan to conduct a pilot deployment on a subset of the TinyGS network to verify the predicted “good-put” gains using real-world pass statistics and actual hardware constraints, moving beyond the empirical models used in simulation.

To extend beyond our optimized scheduling algorithms, we define a lightweight, one-way signaling protocol compatible with TinyGS and similar small satellite networks, which allows each satellite to broadcast its needs to ground stations. A low-power satellite can send a “pause” flag to pause its transmissions and free up receive windows for others, while a data-heavy satellite can send a “high priority” flag to allow stations to allocate additional downlink time. By keeping the link unidirectional (satellite-to-station only), we avoid the licensing and operational hurdles of amateur radio uplinks; amateurs can legally receive, but neither can nor will send commands back to the satellite.

## 8. Conclusions

Allocating space resources to nanosatellites presents a significant challenge for satellite operators, who typically have limited budgets, especially given the increasing demand for transmitting large volumes of data and signals collected by onboard instruments to Earth. This paper presents three heuristics-based algorithms designed to improve the performance of a ground station network for message reception and to maximize the total number of unique messages successfully received by the network. Experimental validation using simulated data shows that the proposed algorithms achieve significant performance improvement. Specifically, they outperform a standard round-robin scheduling algorithm commonly used in such networks, achieving up to a 100% relative increase in the total number of unique messages successfully received under tested scenarios, thereby enhancing the potential for timely data delivery from demanding applications like remote sensing, atmospheric monitoring, and satellite-based IoT services. To sustain these performance gains in operational environments, we further recommend that operators perform periodic recalibration of link probability models to account for hardware degradation and orbital changes.

Building on these promising results, future research can further refine these approaches. We plan to generalize the model to allow inter-satellite communication, which could mitigate ground station visibility constraints by enabling data relay, enabling capable nanosatellites to act as mobile LEO relays with store-and-forward services. In addition, we would like to explore edge-AI (machine learning) algorithms with learnable parameters to directly optimize scheduling decisions based on operational data, further improving the performance of the proposed resource allocation algorithms. Exploring these opportunities aims to significantly enhance data throughput and operational flexibility within dynamic nanosatellite constellations.

## Figures and Tables

**Table 1:** Simulation Control & Satellite Parameters.

Parameter	Value/Description
Simulation Duration	24 h
Simulation Start Date	1 April 2025
Message Transmission Interval	60 s
TLE Update Frequency	60 s
Algorithms Evaluated	Greedy (Base), Cooperative, Pair-Utility, Weighted
Number of Nanosatellites	51
Nanosatellite Transmission Power	0.1–7 W

**Table 2:** Ground Station Network Statistics.

Parameter	Value/Description
Number of Active Stations	92
Network Data Source	TinyGS (Publicly available topology)
Geographic Distribution	Global (North America, Europe, Asia, Australia)
Average Station Spacing	∼50 km (in dense clusters)
Modeled Link Quality ($P_{rx}$)	0.1–1.0 (Derived from station data)
Hardware Characteristics	Implicit in $P_{rx}$ (Empirical/Abstracted)

## Data Availability

The data presented in this study are available on request from the corresponding author. The code is available at https://github.com/kcglab/edgeGS (accessed on 1 December 2025).

## Abbreviations

The following abbreviations are used in this manuscript:

AOS	Acquisition of Signal
CSS	Chirp Spread Spectrum
EOS	Earth Observing System
FSK	Frequency Shift Keying
GSaaS	Ground Station as a Service
HNT	Helium Network Token
IoT	Internet of Things
IoST	Internet of Space Things
LEO	Low Earth Orbit
LoRa	Long Range
MILP	Mixed Integer Linear Programming
NORAD	North American Aerospace Defense Command
SRS	Satellite Range Scheduling
TLE	Two Line Element
